# Oral Hygiene Awareness and Practices among a Sample of Primary School Children in Rural Bangladesh

**DOI:** 10.3390/dj8020036

**Published:** 2020-04-16

**Authors:** Md. Al-Amin Bhuiyan, Humayra Binte Anwar, Rezwana Binte Anwar, Mir Nowazesh Ali, Priyanka Agrawal

**Affiliations:** 1 Centre for Injury Prevention and Research, Bangladesh (CIPRB), House B162, Road 23, New DOHS, Mohakhali, Dhaka 1206, Bangladesh; 2 BRAC James P Grant School of Public Health, BRAC University, 68 Shahid Tajuddin Ahmed Sharani, Mohakhali, Dhaka 1212, Bangladesh; humayra.anwar@bracu.ac.bd; 3 Bangabandhu Sheikh Mujib Medical University (BSMMU) Shahbag, Dhaka 1000, Bangladesh; rezwanabinteanwar@gmail.com (R.B.A.); thebestdentist@yahoo.com (M.N.A.); 4 Department of International Health, Johns Hopkins Bloomberg School of Public Health, 615 N. Wolfe Street, Baltimore, MD 21205, USA; pagrawa6@jhu.edu

**Keywords:** oral health, children, oral hygiene, awareness, rural Bangladesh

## Abstract

Inadequate oral health knowledge and awareness is more likely to cause oral diseases among all age groups, including children. Reports about the oral health awareness and oral hygiene practices of children in Bangladesh are insufficient. Therefore, the objective of this study was to evaluate the oral health awareness and practices of junior school children in Mathbaria upazila of Pirojpur District, Bangladesh. The study covered 150 children aged 5 to 12 years of age from three primary schools. The study reveals that the students have limited awareness about oral health and poor knowledge of oral hygiene habits. Oral health awareness and hygiene practices amongst the school going children was found to be very poor and create a much-needed niche for implementing school-based oral health awareness and education projects/programs.

## 1. Introduction

Oral diseases are a big public health problem all around the world. The prevalence of oral diseases has increased, mainly for individuals from low socio-economic groups [1]. Recently, it has been recognized that oral infection, especially periodontitis, may affect the course and pathogenesis of a number of systemic diseases, such as cardiovascular disease, bacterial pneumonia, diabetes mellitus, and low birth weight [2,3]. Dental caries is one of the major oral diseases for children, which is caused by dental plaque and is directly related with poor oral hygiene practices. [4,5,6,7]. Unfortunately oral hygiene has mostly remained an ignored and unrealized social problem that has steadily become a huge public health burden [8]. Bangladesh is no exception to this issue. Around 46% of 12-year-old children in a study based in rural Bangladesh reported having bleeding gums and calculus deposits on their teeth [9]. Dental caries was also found to be associated with a poor quality of life and low height, weight, and body mass index in previous studies from Bangladesh [5].

Almost all oral diseases need professional dental care; however, due to limited availability or lack of access to oral health services in rural Bangladesh, there is an under-utilization of oral health services among people living in rural areas and those with low income and education [10]. The lack of awareness of self-oral hygiene and its importance also downplays the desire to access oral health services among individuals. It has been reported that the bulk of children from the low socio-economic groups in India have never visited a dentist or been for a dental check-up because of their economic limitations, coupled with lack of awareness, low parental literacy rates, and limited access to oral health care providers [11]. Additionally, multiple studies have showed minimal or non-existent oral hygiene practices in rural areas than in urban cities [12,13].

Oral hygiene is one of the leading standards for maintaining good oral health [14]. The United State department of Health and Human Services stated that no one could be truly healthy unless (s)he is free from the burden of oral and craniofacial diseases and conditions [1]. The negative impacts of poor oral health on daily life reduce chewing performance, constrain food choices, and lead to weight loss, gastro-intestinal disturbances, impaired communication, and low self-esteem and overall well-being [15]. School age is a period of overall development. During this period, the child learns to become a productive member of the peer group [16]. If proper oral hygiene habits are cultivated during this period, habits will go a long way in maintaining the oral health of a child throughout life [17]. So, it is very important to raise awareness about oral health and educate children in this period of life. Daily preventive oral care, with proper brushing and flossing, will help to regress and stop dental problems [18]. It is stated that tooth brushing habits become established during the first few years of childhood and last throughout an individual’s life [19,20].

Imparting knowledge of oral hygiene among children is rural Bangladesh is important to empower them with the skills required to improve their oral hygiene. Given the lack of overarching oral health education programs and facilities to obtain treatment among other barriers to oral health, education for oral health will be beneficial to reduce oral-related morbidity and its economic consequences [21]. Assessing and monitoring the oral hygiene practices and habits of children can easily prevent oral diseases by taking adequate steps in advance, as has been shown in previous studies [22]. Therefore, a study was carried out to evaluate the oral health awareness and hygiene practices of local school children of Mathbaria, Pirojpur, Bangladesh. The aim of this study was to determine the level of oral health awareness and understanding the oral hygiene practices among children 5 to 12 years of age in three schools of rural Bangladesh.

## 2. Materials and Methods

A cross-sectional study was conducted among the junior school students of Mathbaria upazila. A total of 150 school children of 5 to 12 years of age from three purposively selected primary schools of Mathbaria, Pirojpur District, participated in this study. The teachers from each class were requested to assist in data collection and those classes were included where the teacher gave consent. A pre-tested self-constructed questionnaire, developed in the local language, Bangla, was distributed among randomly selected students of those parents who gave consent for their children to participate in the study. The questionnaire included information related to the name and age of the children, education level, and occupation of the parents. (Appendix A). It was further categorized to evaluate the knowledge, practices, and behavior pattern related to oral health. Four dentists involved in the study asked the questions, and the responses were marked according to the answers given by the students. The teachers assisted the students in the completion of the forms. The average time of interview was 10–15 min. The data were analyzed using descriptive and inferential statistics, and the findings were presented in frequencies and charts.

The study protocol was approved by the Institutional Review Board of Bangabandhu Sheikh Mujib Medical University (Reg. No. BSMMU/2017/1976), Bangladesh, dated on 20 August 2017.

## 3. Results

In the present study, a total of 173 students initially agreed to participate in the study. However, 23 students did not complete the questionnaire due to time constraints and their data were dropped in the final analysis. Of the 150 participants that provided complete information and were included in the final analysis, 45.3% were boys and 54.7% were girls. Around 60 (40%) children belonged to households where the head of the family had gone to primary school, 33% had attended secondary school, and 18% completed higher secondary school education and above. The rest of the parents/ caretakers were illiterate or had received a home-based education. For almost all the children, their father was the decision maker. Majority decision makers were involved in agriculture-based work (n = 70, 47%), while 27% (n = 40) were daily laborers. (Table 1).

**Figure 1:**
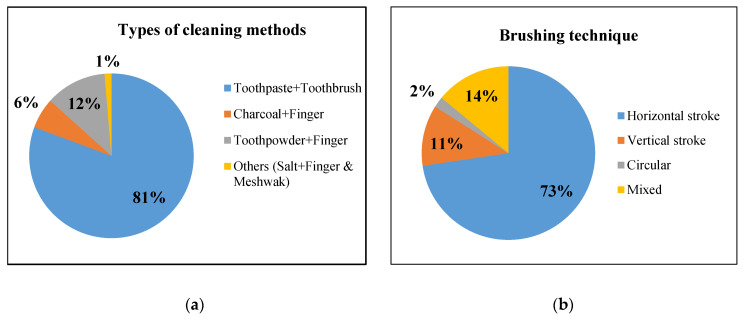
(**a**) Method used to clean the teeth; (**b**) brushing techniques used by students.

The results show that 81% of the children interviewed used a toothbrush and tooth paste to clean their teeth. The other 15% used their finger or neem stick (aka meshwak) and an abrasive such as toothpaste, charcoal, ash and salt. (Figure 1) Around 83% of the students brushed once daily and only 17% brushed twice a day. Most children brushed in the morning, before or after their first meal, while only 8% brushed at night as well before bedtime. When asked about other oral hygiene habits, such as gargling and rinsing oral cavity after meals, only 25% reported carrying out the activity.

Almost 73% of the students brushed their teeth using horizontal strokes (Figure 1b). Among those who used a toothbrush, only five (3.3%) reported using a soft bristled toothbrush and 92 (61.3%) students do not know what bristled toothbrush they should use. Most of them (n = 93, 62%) used a toothpick or stick for inter dental cleaning and around 55% were unaware of when the toothbrush needs to be changed. Only 7% of the students knew that soft tissues of the oral cavity, such as the tongue, also have to be cleaned along with the tooth. Less than one fourth of the students had previously visited a dentist, while others had never visited a dentist (Table 2). Most of the visits were due to pain or dental problems.

## 4. Discussion

In this study, the majority of the school children used a toothbrush and tooth paste to clean their teeth. Almost all children brushed their teeth at least once a day, mostly in the morning before a meal. Awareness of dental floss and interdental brush was lacking. Similar findings for irregular oral hygiene habits have been reported for a school going child population from Jordan [23]. Most children used a toothpick or a stick to clean interdental areas, which are not as effective in cleaning the interdental areas of the tooth when compared with dental floss [24]. Toothpicks are detrimental to the gingival health as well as erupting teeth of children and have been associated with other unintentional puncture wounds [25,26]. The American Dental Association (ADA) recommends that individuals brush twice per day and use floss or other inter dental cleaners once per day to effectively remove microbial plaque [20].

Additionally, 62% of the students were unaware of the frequency of changing toothbrushes. The ADA recommends that toothbrushes be replaced every 3–4 months as the bristles show wear and tear and can harm the surrounding soft tissues structures of the oral cavity. The most commonly used horizontal stroke to clean teeth in this study is among the most detrimental to teeth, causing surface enamel loss, enamel abrasions, as well as contributing to gingival recession [1]. Only 25% of the students rinse their mouth after every meal, and very few knew about tongue cleaning, both of which are simple yet effective measures to reduce food particle deposition and halitosis from the oral cavity [27]. Children were also unaware of the consequences of using hard bristled tooth brushes, which, when combined with faulty tooth brushing techniques, can lead to cervical abrasions, injury to the gums and surrounding soft tissue structures, and teeth sensitivity [28,29,30]. It was also evident from the sociodemographic characteristics of the population that the literacy rate and financial status of the families were low. Hence, it does not come as a surprise that oral hygiene practices and awareness was poor among the sampled children. Studies have associated maternal education with their child’s oral health—mothers with better oral health awareness and positive attitudes towards oral health have children with better oral hygiene practices [31,32]. The poor financial status of the families was most potentially related to limited access to oral health services among these children [31,32,33].

The study had some limitations. Due to the small sample size of the study population, we were not confident in reporting associations between oral health awareness and other socio-demographic characteristics that are known contributors to oral hygiene practices. Additionally, as the study was done in a rural village of Bangladesh, caution needs to be exercised to generalize these findings to other rural and urban areas of the country. However, through the study, we were able to indirectly train the teachers in oral hygiene instructions and proper brushing techniques, as they assisted the research team with explaining the questionnaire and clarifying questions for students.

The findings from our study emphasize the urgent need for oral health education and awareness for school going children to improve their oral health literacy. Studies have shown a reduction in plaque and calculus deposits as well as better gingival health among school children who were exposed to school-based oral health education [34]. Not only would school-based programs improve the oral health of students, the knowledge would get trickled down to parents, teachers, and caregivers, improving the oral health and overall health of communities [33,35,36].

## 5. Conclusions

Oral health awareness and hygiene practices among school going children in rural Bangladesh is very poor. Therefore, school-based oral health awareness and education programs are a necessity to improve the oral health and overall health of children and communities. Furthermore, national oral health behavior data are needed for a national level of planning and an evaluation of health promotion programs.

## Figures and Tables

**Table 1:** Socio-demographic characteristics of the sample population of children.

Characteristics	Total Number	%
**Age group (in years)** 5–7 7–9 9–12	48 59 43	32% 39% 29%
**Gender** Boys Girls	68 82	45.3% 54.7%
Education * No education Primary Secondary Higher secondary and above	14 60 49 27	9% 40% 33% 18%
Occupation * Agriculture Daily laborer Rickshaw/van puller CNG/bus/truck driver Business/shopkeepers Teacher Office worker	70 40 9 3 11 2 15	47% 27% 6% 2% 7% 1% 10%

* Education and occupation of the head of household.

**Table 2:** Oral-health-related awareness among the students.

Variable	Frequency (n)	%
**Brushing frequency**		
Once/daily	121	80.7%
Twice/daily	29	19.3%
**Brushing time**		
Brushing in the morning before meal	133	88.7%
Brushing in the morning after meal	6	4%
Brushing at night after meal	11	7.3%
**Mouth rinsing after meal**		
Yes	37	24.7%
No	113	75.3%
**Types of toothbrush**		
Don’t know	92	61.3%
Soft bristle brush	5	3.3%
Medium/Hard	8	5.3%
Finger	29	19.3%
Meshwak	16	10.7%
**Inter-dental cleaning**		
Toothpick/stick	93	62%
Don’t use	48	32%
Dental floss/dental thread	-	-
Regular thread	9	6%
**How often change the toothbrush**		
Monthly	-	-
3 months	2	1.3%
6 months	13	8.7%
Change when broken	53	35.3%
Don’t know about changing	82	54.7%
**Tongue cleaning/brushing**		
Yes	11	7%
No	16	10.7%
Don’t know	123	82%

## Appendix A

**Basic Questionnaire on Oral Health:**

1. Ne:
2. Age:
3. Sex:
4. Which class do you read in:
5. Bushing frequency
  - Once daily
  - Twice daily
6. Uing of toothbrush and toothpaste
  - Toothpaste + Toothbrush
  - Charcoal + Finger
  - Toothpowder + Finger
  - Others (Salt+Finger & Meshwak)
  - Meshwak (Neem stick)
7. Tpes of toothpaste/powder use?
  - Branded Toothpaste
  - Local toothpaste
  - Tooth powder
  - Coal
  - Salt
  - Meshwak (Neem stick)
8. Brushing time
  - Brushing in the morning before meal
  - Brushing in the morning after meal
  - Brushing at night after meal
9. Brushing technique
  - Horizontal stroke
  - Vertical stroke
  - Circular
  - Mixed
10. Mouth rinsing after meal
  - Yes
  - No
11. Types of toothbrush
  - Soft bristle brush
  - Don’t know
  - Finger
  - Meshwak
  - Medium or Hard
12. Inter-dental cleaning
  - Tooth pick/stick
  - Don’t use
  - Dental floss/dental thread
  - Regular thread
  - Others
13. How often change the toothbrush?
  - Monthly
  - 3 monthly
  - 6 monthly
  - Change when broken
  - Don’t know about changing
14. Tongue cleaning/brushing
  - Yes
  - No
  - Don’t know
15. Brushes tongue by regular toothbrush
  - Yes
  - No
  - Don’t know
16. Do you visit dentist for dental checkup? (if Yes)
  - Yes
  - No
  - Don’t know
17. If yes, then how often?
  - 6 monthly
  - Yearly
  - On pain/if any problem
  - Never
18. Occupation of the parents (Father)
  - Agriculture
  - Daily laborer
  - Rickshaw/van puller
  - CNG/bus/truck driver
  - Business/shopkeepers
  - Teacher
  - Office worker
19. Education of the household parents:
  - No education
  - Primary level
  - Secondary level
  - Higher secondary level and above
